# The association between MTHFR gene polymorphisms (C677T, A1298C) and oral squamous cell carcinoma: A systematic review and meta-analysis

**DOI:** 10.1371/journal.pone.0202959

**Published:** 2018-08-24

**Authors:** Wenzhang Ge, Yang Jiao, Lianzhen Chang

**Affiliations:** 1 Department of Special Clinic, Jinan Stomatological Hospital, Jinan, Shandong, P.R. China; 2 Department of Medicine and Education, Jinan Stomatological Hospital, Jinan, Shandong, P.R. China; 3 Department of Periodontics and Oral Medicine, Jinan Stomatological Hospital, Jinan, Shandong, P.R. China; Charles P. Darby Children's Research Institute, UNITED STATES

## Abstract

A consensus has not been reached regarding the association of MTHFR gene polymorphism and susceptibility to oral squamous cell carcinoma (OSCC). We performed a meta-analysis to better evaluate the association between MTHFR C677T, A1298C polymorphism and OSCC risk. The studies regarding the association of MTHFR C677T, A1298C polymorphisms and OSCC were identified in PubMed and EMBASE and Google Scholar. The pooled odd rates (ORs) with 95%CIs were estimated using a fixed-effect or random-effect model. The associations between MTHFR polymorphisms and OSCC risk were assessed under the dominant, recessive and additive models. A collective total of 1539 OSCC patients and 2131 normal controls were included across 13 studies. The minor T allele of MTHFR C677T was significantly associated with the increased risk of OSCC development(OR = 1.35, 95%CI 1.04–1.76). Individuals carrying the ‘‘T” allele (TT+CT) had a nearly 43% increased risk for OSCC development when compared with CC (OR = 1.43, 95%CI 1.02–1.99). Under additive model, the results also showed that individuals with CT or TT genotype were more susceptible to OSCC than CC (OR = 1.45, 95%CI 1.02–2.08; OR = 1.79, 95%CI 1.28–2.50; respectively). The subgroup analysis by ethnicity revealed that significant difference in C677T allele distribution could be observed in European (OR = 1.33, 95%CI 1.02–1.75) rather than Asian (OR = 1.59, 95%CI 0.91–2.78). No significant association of MTHFR A1298C polymorphism and OSCC risk could be observed. The present study revealed that T allele and TT genotype of MTHFR C677T polymorphism were significantly associated with the increased risk of OSCC development.

## Introduction

Oral squamous cell carcinoma (OSCC) is one of the most lethal cancers worldwide, and is also the most common malignancy of oral cavity accounting for 90–95% of malignant oral tumors[1]. OSCC is characterized by poor prognosis, high recurrence, frequent metastases, with low 5-year survival rates[2]. Until now, OSCC diagnostic procedures have been based mainly on the routine check-up including medical history, intra-oral and extra-oral examination, and standard histology evaluation in case of clinical findings[3]. However, these procedures seem to be inadequate for the prevention or early diagnosis of OSCC based on current epidemiological data[4].

Although the exact etiologic and pathogenetic mechanisms of OSCC remain unclear, several reports suggest that the combination of a susceptible genetic background and environmental factors may initiate and promote the development of OSCC. Except for environmental factors such as tobacco, alcohol, viral infections and inflammation, genetic predispositions have been implicated in oral tumorigenesis and accumulated evidences suggest single nucleotide polymorphisms of some genes may be a vital risk factors for OSCC[5]. Therefore, it has been proposed that detection of individuals at risk for OSCC based on their genetic background such as SNPs could be a promising procedure for early diagnosis, which could lead to greater intervention and follow-up before the appearance of OSCC.

Methylenetetrahydrofolate reductase (MTHFR) plays a vital role in metabolism of folate, which participates in DNA metabolism including DNA methylation, DNA synthesis and repair[6]. Previous studies have indicated that polymorphisms in *MTHFR* gene could affect its functional activity in cancer development[7, 8]. One common polymorphism in the *MTHFR* gene described is C677T. The C677T polymorphism locates in exon 4 at the folate-binding site of the *MTHFR* gene and leads to amino acid substitution (Ala222Val). This substitution could cause thermolabile enzyme with reduced activity[8]. Previous studies have reported C677T polymorphism was associated with susceptibility to several different type of cancers including breast cancer, gastric cancer, oral cancer, etc[9–13]. However, in view of the available data, studies on the association between MTHFR C677T polymorphisms and cancer risk have yielded mixed results. Some reports indicated that T allele could protect against cancer, while another studies revealed that subjects carrying T allele have increased susceptibility to cancer[9–11].

Similar to other cancer, many studies have been performed to investigate the relationship between MTHFR C677T, A1298C polymorphisms and susceptibility to OSCC. However, the results remained inconsistent and controversial. Therefore, we conducted a systematic review and meta-analysis to evaluate the relationship between those two polymorphisms and OSCC risk.

## Material and method

### Data sources and Searches

This systematic review and meta-analysis were carried out based on the reporting guidelines of Meta-analysis of Observational Studies in Epidemiology (MOOSE)[14]. For selection of potential studies, we performed a systematic review of the electronic databases including PubMed, EMBASE, and Google Scholar independently by two authors (W.G and Y.J). Articles published before the end of March 2018 were searched with a combination of mesh term and keywords as follows: oral squamous cell carcinoma, OSCC, methylenetetrahydrofolatereductase, MTHFR, and variant or polymorphism. No restriction on publication language, ethnicity, or geographic region was imposed. All eligible studies were retrieved and their reference lists were also searched for other relevant studies.

### Study selection

The articles were limited to studies on the association between MTHFR gene polymorphism and OSCC. All included studies have to fulfill the following inclusion criteria: (a) a case-control design; (b) the diagnosis of patients should be OSCC; (c) there should be enough data for extraction and evaluation of odds ratio (OR) with 95% confidence interval (CI); Besides, articles would be excluded if they met anyone of the following exclusion criteria: (a) the patients were diagnosed with oral cancers, not OSCC; (b) only one group included, reviews, case reports, mechanism studies as well as non-human studies; (c) insufficient information or data for extraction. When the same or overlapped reports occurred, only the study with largest number of participants was included.

### Data extraction

The required information was independently and in duplicate extracted from all included studies by two authors (W.G and Y.J). A standard reporting form was established to collect the information from all included studies including: first author’s name, country, year of publication, ethnicity, age, typing technique, the total number of OSCC patients and controls, and the frequency of genotype or allele. Besides, Hardy-Weinberg Equilibrium was assessed by using χ2 tests for MTHFR C677T polymorphism in each study. Any disagreements between two authors were resolved by consensus with another authors.

### Assessment of study quality

Due to the lack of a standard quality criteria for meta-analysis of SNP studies, the modified Newcastle-Ottawa scale was used to evaluate the quality of each study[15]. According to this scoring system, each study was assessed based on three criterions: selection of cases and controls, comparability of cases and controls and ascertainment of exposure. NOS scoring system yielded a summary numeric score of quality which ranged from 0 to 9. All included studies could be graded into three categories: low (< = 3), medium (4–6) and high (> = 7) quality [16].

### Statistical analyses

The pooled ORs with 95%CIs were calculated in a fixed-effect or random-effect model to assess the strength of the association between MTHFR polymorphism and susceptibility to OSCC. The χ2-based Q test and I2 statistic were used to judge the heterogeneity across included studies. For Q test, P<0.10 was considered to be representative of statistically significant heterogeneity, and I2 statistic represented the percentage of total variation contributed by a between-study variation ranged from 0% to 100%[17]. If significant heterogeneity was observed, a random-effect model would be used to pool data. Otherwise, a fixed-effect model was used. The publication bias was determined by using funnel plots, Egger’s test and Begg’s test. Sensitivity analysis was performed to examine data stability and evaluate the impact of each individual study on the final ORs [18]. All analyses were conducted using Comprehensive meta-analysis software V.3.0(BiostatInc, Englewood Cliffs, New Jersey, USA). P value of less than 0.05 was considered significant.

## Result

### Source study

**Fig 1:**
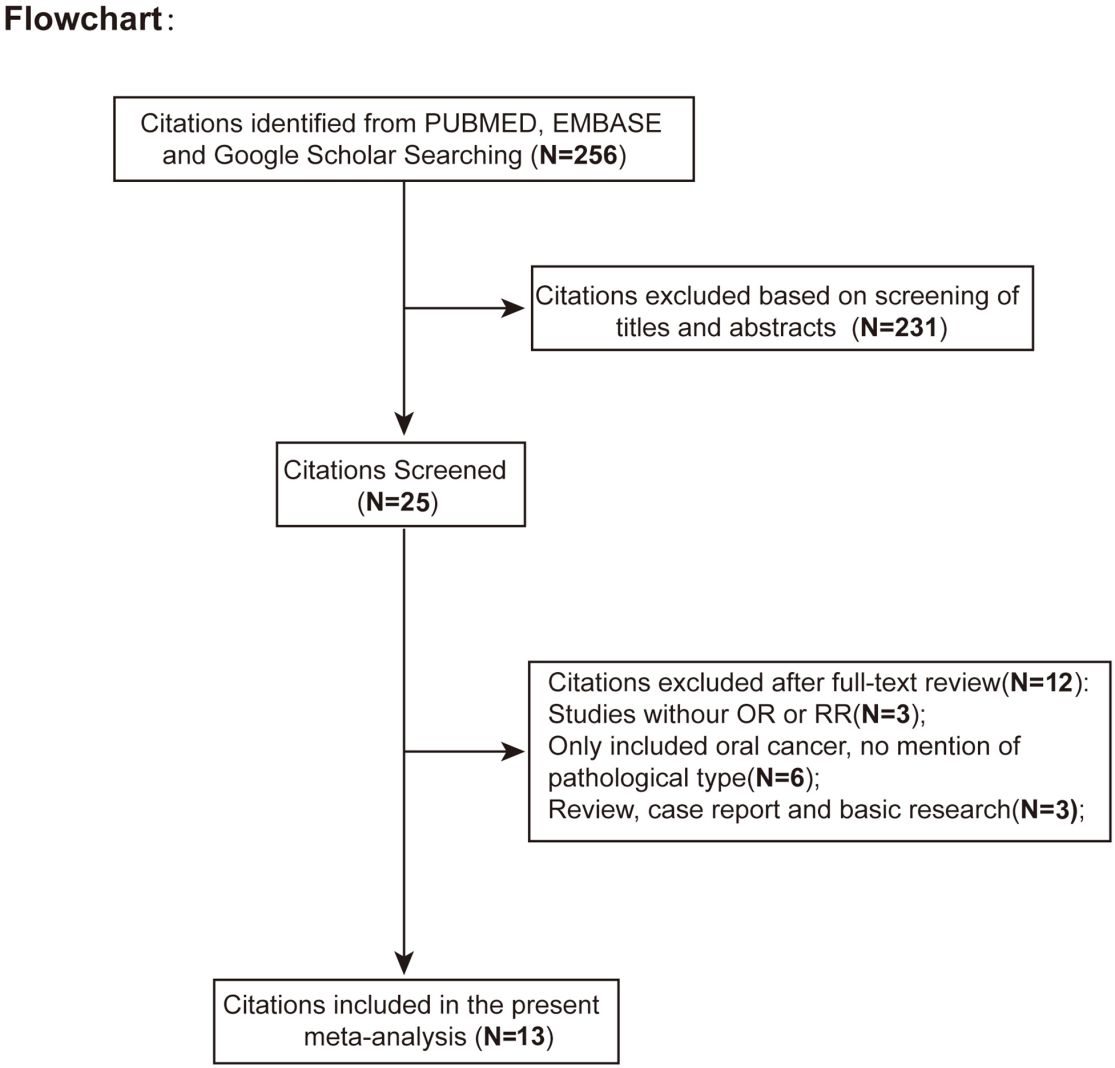
The flowchart showing articles identification, inclusion and exclusion. 13 eligible studies were included.

The derivation of the source studies included in the present study was presented in Fig 1. From the initial search, a total of 256 papers were identified. After screening of abstracts or titles, 231 articles were excluded. 25 full-text articles were retrieved and assessed for eligibility. 12 articles were excluded based on exclusion criteria, leaving 13 eligible studies with MTHFR polymorphisms and susceptibility to OSCC for final inclusion in the present meta-analysis[19–30].

### Study characteristic

All these 13 articles have been published in English. The information and characteristics from each study were described in Table 1. A collective total of 1539 OSCC patients and 2131 normal controls were included across these 13 studies, which were conducted in population samples from 5 European background and 5 Asians background as well as 3 South American background. Sample sizes of these studies ranged from 119 to 700. 9 out of these 13 studies have stated that OSCC patients were diagnosed based on histopathological confirmation[19–23, 26–28]. MTHFR gene polymorphisms were determined by polymerase chain reaction-restriction fragment length polymorphism (PCR-RFLP) method in all included studies. Hardy-Weinberg equilibrium tests indicated that deviation from HWE could been observed in some studies (Table 1). The results from quality assessment revealed that 6 studies scored 8 stars, 6 studies scored 7 stars, 1 study scored 6 stars (S1 Table).

**Table 1:** Characteristic of 13 included studies in the present study.

Author	Year	Country	Ethnicity	Case/Control	Diagnosis Criteria	Genotyping Method		Genotype		Allele			HWE	
								OSCC	Control		OSCC	Control	OSCC	Control
MTHFR C677T														
Solomon PR, et al	2008	India	Asian	126/100	NS	PCR-RFLP	TT	23	10	T	101	62	0.301	0.855
							CT	55	42	C	151	138		
							CC	48	48					
Supic G, et al	2011	Serbia	European	96/162	NS	PCR-RFLP	TT	14	16	T	60	98	0.09	0.66
							CT	32	66	C	132	226		
							CC	50	80					
Vylliotis A, et al	2013	Greece	European	110/120	Histopathological Diagnosis	PCR-RFLP	TT	6	10	T	88	85	<0.01	0.04
							CT	76	65	C	132	155		
							CC	28	45					
Bezerra AM, et al	2014	Brazil	South American	32/90	Histopathological Diagnosis	PCR-RFLP	TT	1	1	T	14	43	0.58	0.02
							CT	12	41	C	50	137		
							CC	19	48					
Miri-Moghaddam E, et al	2015	Iran	Asian	57/62	Histopathological Diagnosis	PCR-RFLP	TT	2	1	T	25	16	0.57	0.97
							CT	21	14	C	89	108		
							CC	34	47					
Addala L, et al	2013	India	Asian	150/150	Histopathological Diagnosis	PCR-RFLP	TT	2	1	T	28	10	0.50	0.03
							CT	24	8	C	272	290		
							CC	124	141					
Bektas-Kayhan K, et al	2014	Turkey	European	108/116	Histopathological Diagnosis	PCR-RFLP	TT	7	5	T	57	53	0.80	0.58
							CT	43	43	C	159	179		
							CC	58	68					
Sailasree R, et al	2011	India	Asian	101/139	Histopathological Diagnosis	PCR-RFLP	TT	1	1	T	10	31	0.11	0.53
							CT	8	29	C	192	245		
							CC	92	108					
Vairaktaris E, et al	2006	Greece	European	110/120	Histopathological Diagnosis	PCR-RFLP	TT	6	10	T	88	85	<0.01	0.04
							CT	76	65	C	132	155		
							CC	28	45					
Barbosa A, et al	2016	Brazil	South American	101/102	Histopathological Diagnosis	PCR-RFLP	TT	6	11	T	57	63	0.31	0.55
							CT	45	41	C	145	141		
							CC	50	50					
Naqvi H, et al	2016	India	Asian	350/350	NS	PCR-RFLP	TT	20	7	T	108	47	0.11	<0.01
							CT	68	33	C	592	653		
							CC	262	310					
Ferlazzo N, et al	2017	Italy	European	58/90	NS	PCR-RFLP	TT	18	15	T	70	68	0.10	0.33
							CT	34	38	C	46	112		
							CC	6	37					
Galbiatti ALS, et al	2012	Brazil	South American	130/531	Histopathological Diagnosis	PCR-RFLP	TT/CT	81	305	T	NA	NA	NA	NA
							CC	49	226	C	NA	NA		
MTHFR A1298C														
Sailasree R, et al	2011	India	Asian	101/139	Histopathological Diagnosis	PCR-RFLP	AA	37	46	A	148	151	0.06	0.08
							AC	74	59	C	112	137		
							CC	19	34					
Barbosa A, et al	2016	Brazil	South American	101/102	Histopathological Diagnosis	PCR-RFLP	AA	60	53	A	156	150	0.89	0.27
							AC	36	44	C	46	54		
							CC	5	5					
Ferlazzo N, et al	2017	Italy	European	58/90	NS	PCR-RFLP	AA	32	57	A	84	139	0.29	0.06
							AC	20	25	C	32	41		
							CC	6	8					
Galbiatti ALS, et al	2012	Brazil	South American	130/531	Histopathological Diagnosis	PCR-RFLP	AA	49	316	T	NA	NA	NA	NA
							AC/CC	81	215	C	NA	NA		

NS: None Stated

### MTHFR C677T polymorphism and susceptibility to OSCC

**Fig 2:**
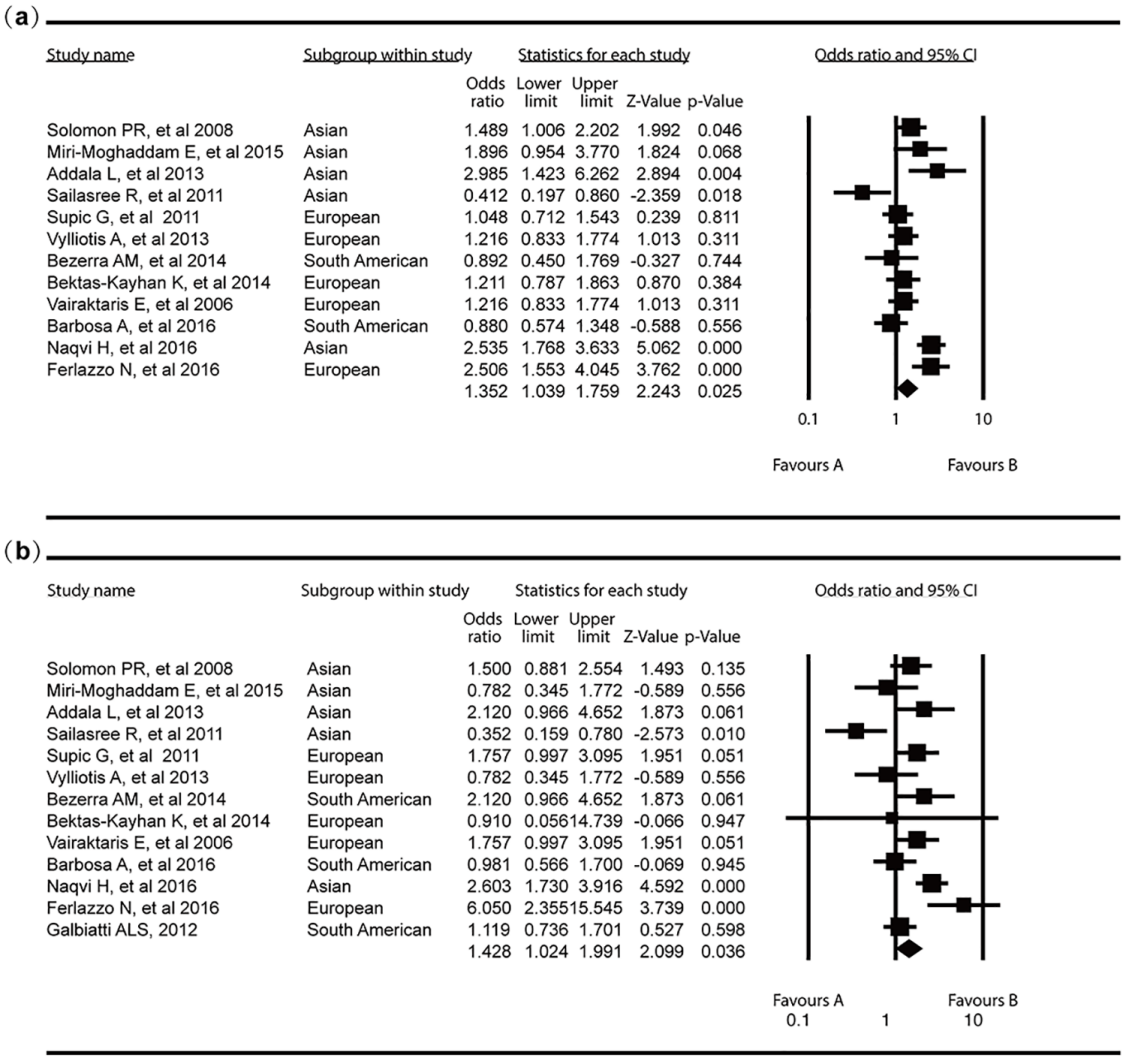
Forest plots for the association between MTHFR C677T polymorphism and OSCC risk in overall population. (a) T vs C; (b) TT vs CC.

A summary of the meta-analysis for MTHFR C677T polymorphism and OSCC risk was presented in Table 2. The pooled data showed the frequency of minor T allele was significantly higher in OSCC patients than controls, suggesting that this allele may contribute to OSCC development(OR = 1.35, 95%CI 1.04–1.76; Fig 2a). Heterogeneity assessment demonstrated that significant inter-study variation existed in the meta-analysis (P<0.01, I^2^ = 73.0%).

**Table 2:** Meta-analysis of associations between MTHFR C677T, A1298C polymorphisms and OSCC risk using the additive, dominant, recessive model.

SNP	Comparison	OR with 95%CI	Heterogeneity			Publication Bias	
			χ2	Q test	I^2^	Begg	Egger
C677T							
Overall	**T vs C**	**1.35 (1.04–2.24)**	41.4	<0.01	73.4%	0.83	0.34
	TT vs CT	1.14 (0.82–1.58)	10.6	0.48	0.00%	0.63	0.94
	**TT vs CC**	**1.79 (1.28–2.50)**	16.5	0.123	33.5%	0.73	0.98
	**CT vs CC**	**1.45 (1.02–2.08)**	40.2	<0.01	72.1%	0.78	0.93
	TT vs CT+CC	1.40 (0.73–2.48)	37.1	<0.01	17.3%	0.58	0.53
	**CT+TT vs CC**	**1.43 (1.02–1.99)**	37.3	<0.01	70.5%	0.83	0.44
Asian	T vs C	1.59 (0.91–2.78)	21.6	<0.01	81.0%	0.62	0.52
	TT vs CT	1.55 (0.87–2.78)	0.91	0.92	0.00%	1.00	0.83
	**TT vs CC**	**2.65 (1.51–4.64)**	0.75	0.95	0.00%	0.62	0.42
	CT vs CC	1.52 (0.76–3.03)	21.4	<0.01	81.5%	0.62	0.54
	**TT vs CT+CC**	**2.44 (1.42–4.21)**	1.07	0.90	0.00%	0.62	0.81
	CT+TT vs CC	1.23 (0.63–2.43)	22.8	<0.01	82.1%	0.14	0.14
European	**T vs C**	**1.33 (1.02–1.75)**	8.76	0.07	54.5%	0.22	0.07
	TT vs CT	1.06(0.69–1.64)	5.60	0.23	29.3%	0.46	0.32
	TT vs CC	1.72 (0.85–3.50)	9.06	0.06	55.8%	0.80	0.82
	CT vs CC	1.64 (0.97–2.78)	26.2	<0.01	84.8%	0.08	0.10
	TT vs CT+CC	1.12 (0.38–3.85)	4.67	0.458	0.00%	1.00	0.53
	CT+TT vs CC	1.82 (0.99–3.33)	10.6	0.03	26.1%	0.80	0.98
A1298C							
Overall	C vs A	0.91 (0.71–1.17)	6.08	0.05	67.1%	0.60	0.57
	CC vs AA	0.68 (0.30–1.54)	0.22	0.90	0.00%	0.60	0.94
	CC vs AC	0.64 (0.35–1.19)	0.73	0.70	0.00%	0.12	0.15
	AC vs CC	1.06 (0.74–1.51)	6.91	0.03	71.0%	0.60	0.79
	CC vs AC+AA	0.66 (0.27–1.58)	0.05	0.97	0.00%	0.60	0.67
	CC+AC vs AA	1.22 (0.65–2.26)	4.70	<0.10	81.7%	0.12	0.78

**Fig 3:**
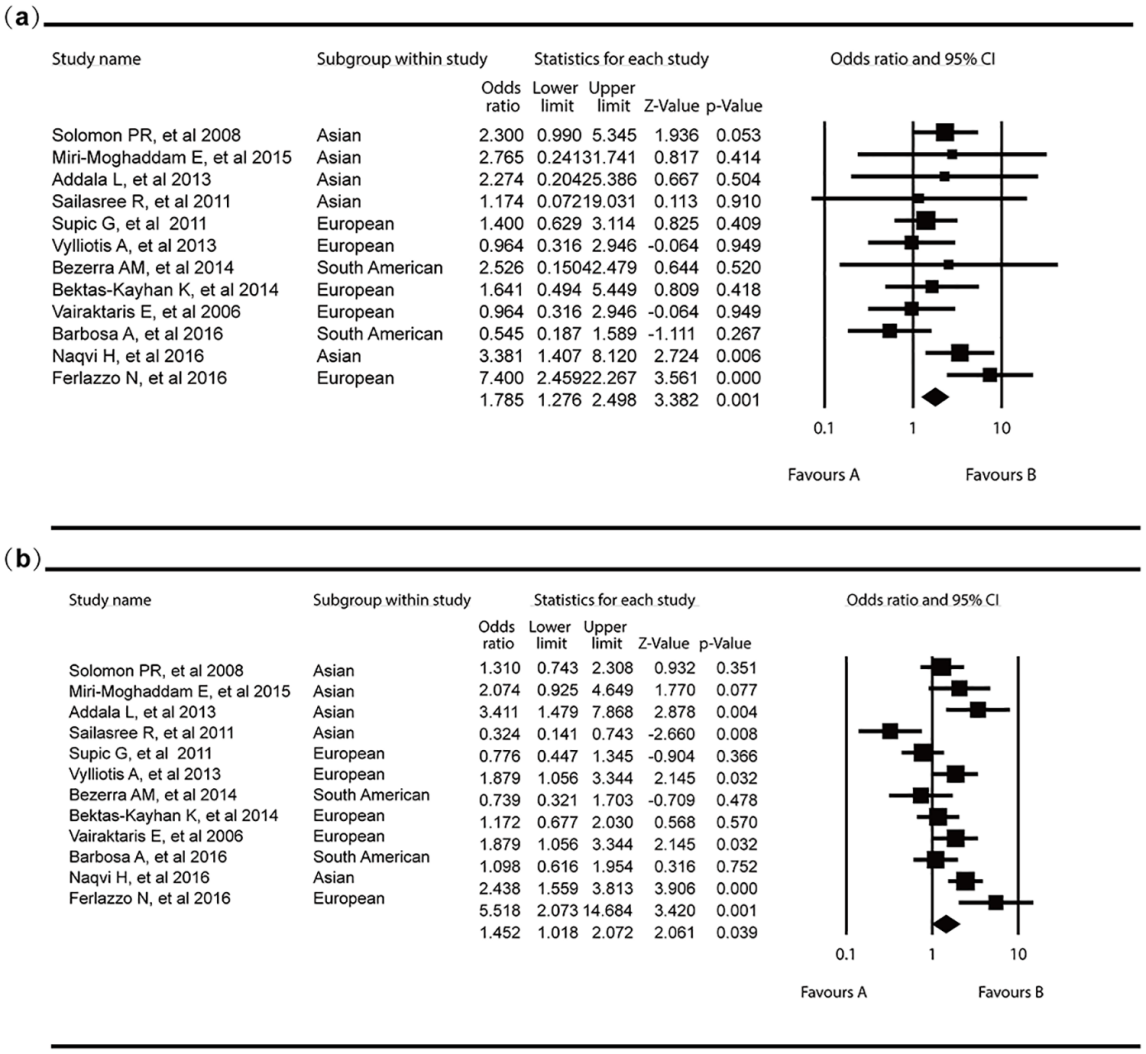
Forest plots for the association between MTHFR C677T polymorphism and OSCC risk in overall population. (a) CT vs CC; (b) CT+TT vs CC.

The additive, dominant and recessive models of inheritance were also used to assess the association between reported MTHFR C677T polymorphism and OSCC risk. Individuals carrying ‘‘T” allele (TT+CT) had a nearly 43% increased risk for the development of OSCC when compared with CC homozygotes (OR = 1.43, 95%CI 1.02–1.99; Fig 2b). The TT genotype was associated with a high risk for OSCC, when compared with the C allele (CC+CT) homozygote (OR = 1.40, 95%CI 0.73–2.48), but without statistical difference. Under additive model, the results showed that individuals with TT or CT genotype were more susceptible to OSCC than CC homozygotes (OR = 1.79, 95%CI 1.28–2.50; OR = 1.45, 95%CI 1.02–2.08; respectively, Fig 3a and 3b). However, the results showed that individuals with TT, CT genotype have no significant difference (Table 2). Heterogeneity analyses revealed significant inter-study variation under dominant recessive and additive modes.

### Subgroup meta-analysis stratified by ethnicity

The subgroup meta-analyses by ethnicity were further conducted (Table 2). The results indicated that significant difference in C677T allele distribution could be discovered in European (OR = 1.33, 95%CI 1.02–1.75) rather than Asian (OR = 1.59, 95%CI 0.91–2.78). In Asian population, we also found individuals with TT homozygote have significantly more susceptibility to OSCC than those with CT or CC (OR = 2.44, 95%CI 1.42–4.21). And the pooled ORs showed that individuals who carried TT homozygote have a 165% increased risk of OSCC compared with those with CC homozygote (OR = 2.65, 95%CI 1.51–4.64). In European population, no significant difference could be observed under the additive, dominant and recessive models.

### MTHFR A1298C polymorphism and susceptibility to OSCC

4 studies including 369 OSCC cases and 862 normal controls have discussed the association of MTHFR A1298C polymorphism and susceptibility to OSCC (Table 1). No significant association of MTHFR A1298C polymorphism and OSCC risk could be observed (Table 2). Due to smaller sample size, subgroup meta-analysis could not be performed.

### Sensitivity analysis

Sensitivity analysis was carried out to evaluate the influence of each individual study on the overall OR. The stability and reliability of overall ORs were examined using the leave-one-out method, which repeated the analysis after sequential exclusion of each study. The significant association of the minor T allele at C677T polymorphism and OSCC risk disappeared after excluding Solomon PR’ study or Naqvi H’ study, which may be due to small sample size and different ethnicity. Besides, the results were relatively constant and stable.

### Funnel plots

**Fig 4:**
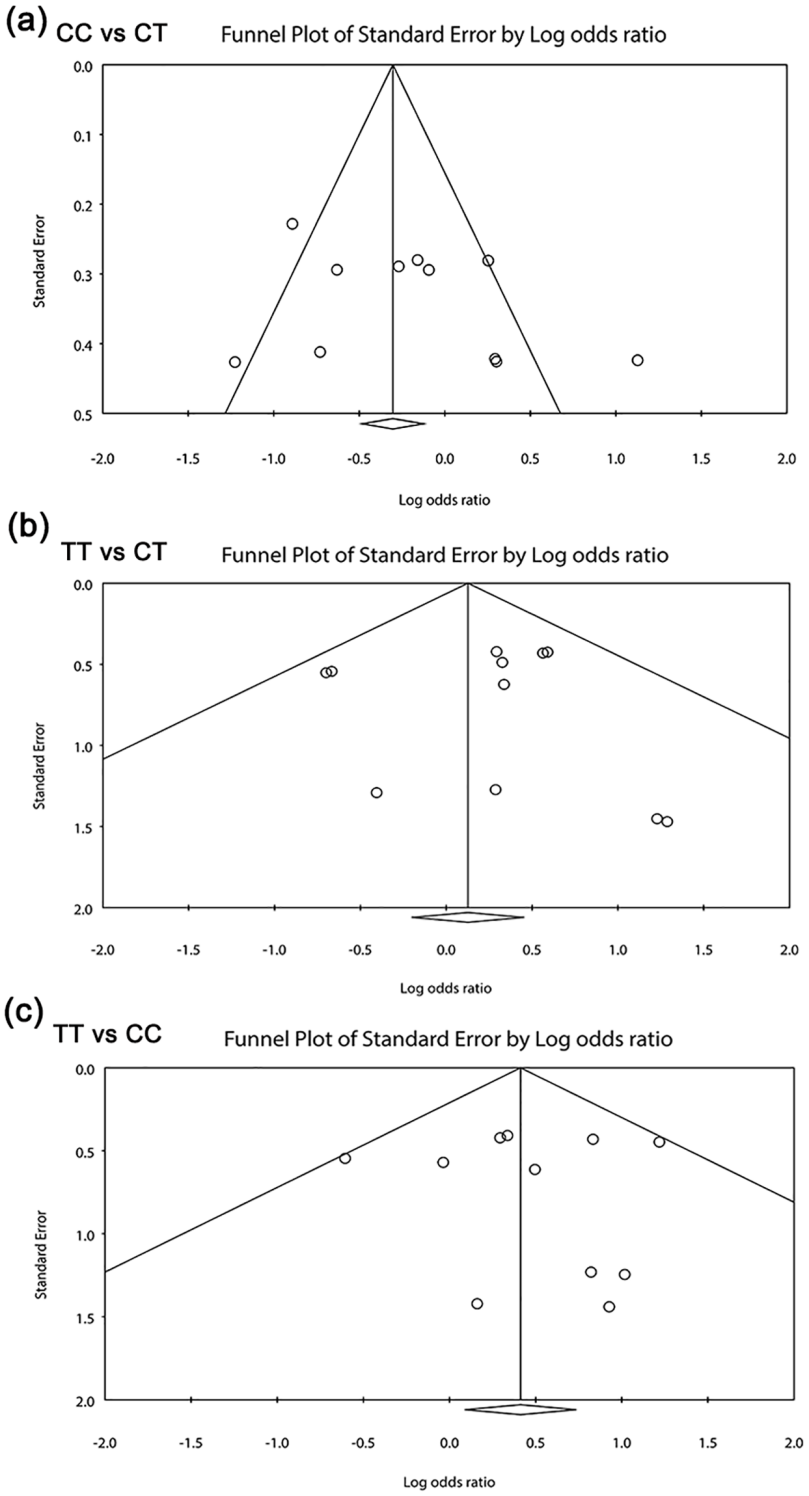
Funnel plots of the meta-analysis for the association of MTHFR C677T polymorphism and OSCC risk under additive models. (a) CC vs TC; (b) TT vs Ct; (c) TT vs CC.

**Fig 5:**
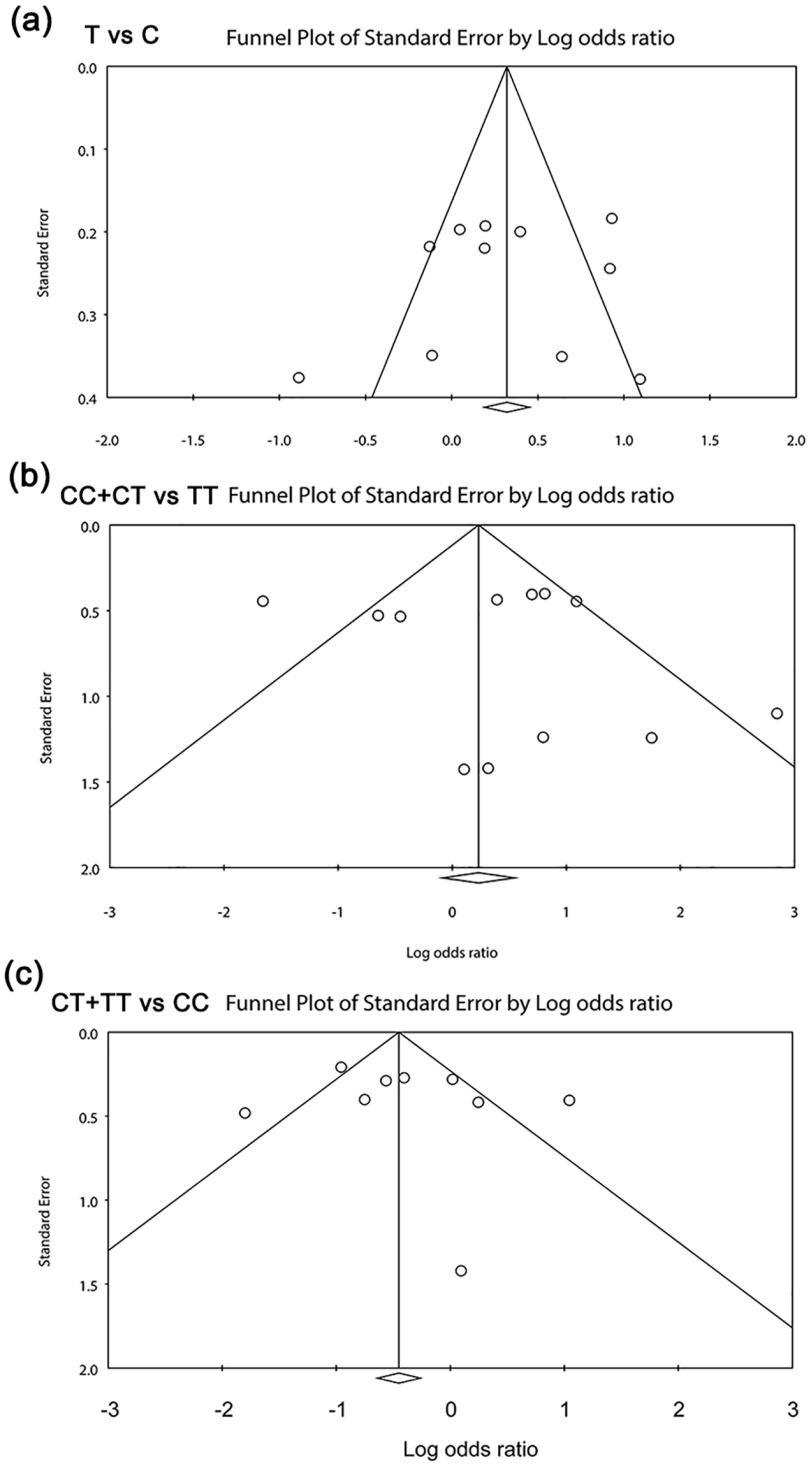
Funnel plots of the meta-analysis for the association of MTHFR C677T polymorphism and OSCC risk under allele, dominant, recessive models. (a) T vs C; (b) CC+CT vs TT; (c) TT+CT vs CC.

The funnel plots for association between MTHFR C677T, A1298C polymorphisms and OSCC risk were drawn, which also did not reveal visually significant publication bias (Figs 4 and 5). As expected, both Egger’s test and Begg’s test demonstrated no publication bias (Table 2).

## Discussion

MTHFR is a key enzyme in the folate metabolism process, it could catalyze the conversion of 5,10-methylenetetrahydrofolate acid into 5-methyltetrahydrofolate acid which is a major circulating form of folate acid[6]. Accumulated evidences suggested MTHFR C677T and A1298C had functional relevance to alter the enzyme activity and cause the alteration of amino acid and thereby a decreased plasma folate concentration[31]. Deficiency of folate could result in DNA strand breaks, damaged DNA methylation and DNA repair. Therefore, these polymorphism may cause carcinogenesis. It also remains of interest to evaluate whether MTHFR polymorphism was involved in the development of cancer. However, the effects of MTHFR polymorphism on cancer risk were controversial. Recent published meta-analysis demonstrated MTHFR C677T polymorphism has an increased risk of breast and esophageal cancer, while a decreased risk of colorectal cancer was detected[9–11]. Thus, MTHFR polymorphism might play various roles in different type of cancers. In the present study, we conducted a systematic review and meta-analysis to determine the effect of MTHFR C677T and A1298C polymorphisms on OSCC risk. The significant association between minor T allele, homozygote TT of C677T polymorphism and OSCC risk were observed, suggesting MTHFR C677T polymorphism was associated with increased susceptibility to OSCC. However, no significant association between MTHFR A1298C polymorphisms and OSCC risk could be observed.

Reports of MTHFR C677T polymorphism as a carcinogen for multiple cancers promote many investigations to explore its genetic effects on oral cancer. The genetic association of C677T polymorphism and oral cancer risk was originally investigated in Weinstein’s study, but no significant association was implicated. Although several continued effects have been placed to the replication of the initial finding, the following studies yielded controversial results. Due to the limitation of study size, the single studies may lack sufficient statistical power to obtain a precise conclusion[32]. In 2014, Jia has conducted a meta-analysis to evaluate the overall risk of MTHFR C677T in oral cancer, which showed a marginal association of MTHFR C677T polymorphism and oral cancer risk that individuals with CT have a 14% decreased risk for oral cancer compared with CC[33]. OSCC is the most common type of oral cancer, several studies have also focused on the role of MTHFR C677T polymorphism in OSCC. Similarly, the contribution of C677T polymorphism in OSCC has been investigated in different cohort with conflicting results. Thus, we conducted this meta-analysis to evaluate whether the C677T polymorphism confers susceptibility to OSCC. We found T allele contributed to the higher risk of OSCC compared to C allele. Meanwhile, the result found individuals with TT genotype have an increased risk of OSCC compared with CC genotype. In recessive mode, the result revealed that individuals carrying T allele (CT+TT) have more susceptibility to OSCC when compared with CC genotype. The result was not in consistent with Jia’s conclusion that CT genotype was associated with a decreased risk of oral cancer, our result showed a 45% increased risk of developing OSCC compared with CC genotype. The difference may attribute to the inclusion study. All oral cancer could be included in Jia’s study, while our study only included OSCC patients. 3 out of 7 studies included in Jia’s study were excluded in the present study because they did not meet the OSCC diagnosis. After including more new reports, a total of 13 papers were included in the present study, we thought this study could provide more accurate conclusion based on more sample size, and strict inclusion criteria. Considering the possible effects of the confounding factors on the overall data, we conducted subgroup analyses stratified by ethnicity. Compared with overall data, significant association between C677T allele with OSCC risk was only observed in European population. While the significant association under TT vs CC, TT vs TC+CC could only be observed in Asian population, and the ORs were much higher than those in overall population. After stratified by ethnicity, the heterogeneity could be significantly reduced in both groups. Therefore, the different ethnicities may contribute to the high degree of heterogeneity. As for other confounders such as age, gender, smoking status or virus infection, we could not assess the effect of these confounders on the heterogeneity due to insufficient primary data.

Except for polymorphism, previous studies have demonstrated that epigenetic modification of *MTHFR* gene such as gene-specific DNA methylation was implicated in several cancer etiology. For example, significantly higher frequency of MTHFR methylation were observed in HPV-positive cervical cancer than HPV-negative cervical cancer or normal controls[34]. Meanwhile, due to low folate deficiency resulting from MTHFR polymorphism could affect DNA methylation through mediating the transfer of one-carbon moieties, MTHFR polymorphism have been reported to be associated with the hypermethylation of several cancer-related genes. In OSCC patients, a significant association between MTHFR gene polymorphisms and p16, MGMT gene promoter methylation have been found[29]. In gastric cancer and esophageal squamous cell carcinoma, the previous study also observed that individuals with homozygotes (TT) of MTHFR C677T polymorphism had significant risk of hypermethylation of MGM, hMLH1, P16[35–37].

Folate deficiency resulting from MTHFR polymorphism could impair the DNA repair function for chromosome damage caused by environmental factor such as alcohol, smoking, HPV virus, while folate intake could also reduce the risk of oral and pharyngeal cancers[38]. Meanwhile, previous study have demonstrated that high folic acid consumption could reduce MTHFR protein and activity, creating a pseudo-MTHFR deficiency[39]. Combined with the important role of MTHFR in the folate metabolism process, the hypothesis of gene-environmental factor interaction in carcinogenesis has been purposed. For example, MTHFR 677 TT genotype was found to have high risk association with heavy drinking population (>396g ethanol/week) rather than light drinking population(<198g ethanol/week)[24]. The risk for multiple methylation was also significantly increased in heavy-drinking patients with TT genotype compared with CC and CT genotype(20940365). Similarly, concomitant use of alcohol, cigarette and possessing T allele of C677T were significantly higher in OSCC patients[21]. Besides, the investigators have found homozygotes (TT) of C677T with low levels of folate were significantly associated with decreased methylation of MGMT in Chinese glioma patients[40]. Low vitamin B2 and low methionine intake were significantly associated with an increased risk of colorectal tumor in individuals with homozygotes (TT) of C677T [41]. However, some studies demonstrated no overall association in breast cancer patients[42]. Therefore, these findings, as well as significant interactions between MTHFR polymorphisms and environmental triggers, or nutrients warrant further investigation.

Of course, several limitations might be involved in the present study. First, the number of eligible studies was relatively less, limiting the ability to get firm conclusion. Second, only studies written in English were searched and included. It is possible that publications of other languages that might meet the inclusion criteria were missed. Thus, even though the publication bias tests did not yield any significant publication bias, it is impossible to exclude completely the confounders due to less number of included studies and the fact that negative results are commonly not published. Third, OSCC patients from 9 included studies were pathological diagnosed, while diagnosis criteria was not described in another 4 studies. Although the overall ORs were relatively constant and stable after excluded these 4 papers (data not shown), we could not completely exclude the influence of this factor on the conclusion. Forth, subgroup analyses regarding age, gender, smoking status or virus infection could not be performed. Therefore, future investigations with large sample sizes and detailed characteristics were required, such as genome-wide association studies in different ethnic cohort of OSCC patients.

In conclusion, the present study observed significant association between MTHFR C677T polymorphism and OSCC risk. T allele and TT genotype was identified to be associated with the increased risk for OSCC. Also, individuals with the T allele (CT+TT) have more susceptibility to OSCC than those with CC homozygotes. Besides, no significant association between MTHFR A1298C polymorphism could be observed.

## Supporting information

S1 Table Methodological quality of Case-control Studies according to the NEWCASTLE-OTTAWA quality assessment scale. (DOC)

S2 Table PRISMA-checklist of the present meta-anlsysis. (DOCX)

S3 Table Raw-data of the present study. (XLSX)
