# Supplementary material for: The association between MTHFR gene polymorphisms (C677T, A1298C) and oral squamous cell carcinoma: A systematic review and meta-analysis
Source: PLoS One. 2018 Aug 24;13(8):e0202959. doi: 10.1371/journal.pone.0202959 (PMC6108503; doi:10.1371/journal.pone.0202959)
Supplement: S1 Table — (DOC) [file pone.0202959.s001.doc]

S1Table. Methodological quality of Case-control Studies according to the NEWCASTLE-OTTAWA Quality Assessment Scale

| **Study** | **Selection** | | | | **Comparability** | **Exposure** | | | **Total** |
| --- | --- | --- | --- | --- | --- | --- | --- | --- | --- |
|  | **Is the case definition adequate** | **Representativeness**  **of the cases** | **Selection of**  **controls** | **Definition of**  **controls** | **Comparability of cases and controls on the basis of the design or analysis** | **Ascertainment of exposure** | **Same method of ascertainment for cases and controls** | **Non-Response rate** |  |
| Solomon PR, et al | ★ | / | ★ | ★ | ★ | ★ | ★ | ★ | **7 Stars** |
| Supic G, et al | ★ | / | ★ | ★ | ★ | ★ | ★ | ★ | **7 Stars** |
| Vylliotis A, et al | ★ | ★ | ★ | ★ | ★ | ★ | ★ | ★ | **8 Stars** |
| Bezerra AM, et al | ★ | / | ★ | ★ | ★ | ★ | ★ | / | **6 Stars** |
| Miri-Moghaddam E, et al | ★ | ★ | ★ | ★ | ★ | ★ | ★ | ★ | **8 Stars** |
| Addala L, et al | ★ | ★ | ★ | ★ | ★ | ★ | ★ | ★ | **8 Stars** |
| Bektas-Kayhan K, et al | ★ | / | ★ | ★ | ★ | ★ | ★ | ★ | **7 Stars** |
| Sailasree R, et al | ★ | ★ | ★ | ★ | ★ | ★ | ★ | ★ | **8 Stars** |
| Vairaktaris E, et al | ★ | ★ | ★ | ★ | ★ | ★ | ★ | ★ | **8 Stars** |
| Barbosa A, et al | ★ | ★ | ★ | ★ | ★ | ★ | ★ | ★ | **8 Stars** |
| Naqvi H, et al | ★ | / | ★ | ★ | ★ | ★ | ★ | ★ | **7 Stars** |
| Ferlazzo N, et al | ★ | / | ★ | ★ | ★ | ★ | ★ | ★ | **7 Stars** |
| Galbiatti ALS, et al | ★ | / | ★ | ★ | ★ | ★ | ★ | ★ | **7 Stars** |
